# 

**Published:** 1864-07

**Authors:** 


### CONTENTS.

### ORIGINAL CONTRIBUTIONS.

ART. XXV. REPORT OF SPECIAL COMMITTEE ON DISEASES OF THE EYE. By E. L. HOLMES, M.D., of Chicago, .................... 385
ART. XXVI. CEREBRO-SPINAL MENINGITIS. Letter read before the Chicago Medical Society. By JOHN BARTLETT, M.D., from DR. E. Y. YAGER, of Chillicothe, Livingston Co., Mo.,.................... 395
ART. XXVII. CEREBRO-SPINAL MENINGITIS. By DR. MCVEY, of Morgan Co., Ill.,.................... 403
ART. XXVIII. NOTES OF A CASE OF CEREBRO-SPINAL MENINGITIS. By D. W. YOUNG, M.D., of Aurora, Ill.,.................... 405
ART. XXIX. HISTORY OF THE SYMPTOMS OF J. P. F., PREVIOUS TO DEATH, by Letter from Dr. CLARK; Also, SYNOPSIS OF NOTES TAKEN AT THE POST MORTEM. By HIRAM WANZER, M.D., of Chicago, Ill. 410
ART. XXX. SUB-CUTANEOUS INJECTION OF QUININE FOR THE CURE OF INTERMITTENT FEVER.—WHO IS ENTITLED TO PRIORITY IN THE PRACTICE? By IGNATIUS LANGER, M.D., of Davenport, Iowa........ 413

### SELECTIONS.

Cases of Gastralgia and Irritable Stomach; Use of the Purified Oxide of Manganese.................... 414
On a New Operation for Obtaining Union of an Ununited Fracture, with Remarks on its Application in certain cases of Recent Fracture. By E. R. Rickersteth, F.R.C.S., Surgeon to the Liverpool Royal Infirmary.................... 416
On Dreaming and Somnambulism in Relation to the Functions of Certain Nerve-Centres. By W. S. Savory, Esq., F.R.S.................... 422
Specialties.................... 428

### EDITORIAL.

Record of Proceedings of the American Medical Association. Fifteenth Annual Meeting.................... 430
Semi-Annual Meeting of the Esculapian Society.................... 442
Dr. Langer, of Davenport, on Sub-Cutaneous Medication.................... 442
Book Received. A Treatise on Chronic Inflammation,.................... 443
Foreign Intelligence.................... 443
Medical News.................... 444
The Cinchona Plant in Jamaica.................... 444

